# Perceptions of benefits, challenges, and utility of simulation-based education among medical faculty of Khyber Pakhtunkhwa, Pakistan

**DOI:** 10.12669/pjms.41.11.12263

**Published:** 2025-11

**Authors:** Munir Ahmad Khan, Nowshad Asim, Brekhna Jamil, Mehreen Iqbal

**Affiliations:** 1 Munir Ahmad Khan, MBBS, FCPS, MHPE, Assistant Professor of Medicine and Chairperson Medical Education Department. Gomal Medical College, MTI, Dera Ismail Khan, Khyber Pakhtunkhwa, Pakistan; 2 Nowshad Asim, MBBS, DFM, MHPE, PhD Scholar Assistant Professor of Medical Education., Institute of Health Professions Education and Research, Khyber Medical University, Peshawar, Khyber Pakhtunkhwa, Pakistan; 3 Brekhna Jamil, BDS, MPH, MHPE, PhD (UK) Professor of Medical Education., Institute of Health Professions Education and Research, Khyber Medical University, Peshawar, Khyber Pakhtunkhwa, Pakistan; 4 Mehreen Iqbal, MBBS, M.Phil, FCPS, MRCOG, MHPE. District Headquarter (DHQ) Hospital, Khyber District, Health Department, Khyber Pakhtunkhwa, Pakistan

**Keywords:** Faculty training, Implementation challenges, Simulation-based education, Skill acquisition, Perceptions, Medical faculty

## Abstract

**Objectives:**

To explore the perceived benefits and uses of simulation-based education (SBE) among medical faculty and to identify the challenges they encounter in its use and implementation.

**Methodology:**

This qualitative case study employed a qualitative design to explore the perceptions of medical faculty regarding simulation-based education (SBE) at three medical colleges in Pakistan. This study was conducted at Khyber Medical University, Peshawar from April to September of 2024. Using semi-structured interviews, the study collected data from 10 faculty members selected through convenience sampling. The interviews were guided by the Consolidated Framework for Implementation Research (CFIR) and were face-to-face or via phone, ensuring participant comfort and consent. Data analysis involved coding and thematic analysis, manually and using Otter.ai software.

**Results:**

The thematic analysis identified six key themes related to simulation-based learning (SBL) in medical education: (1) Advantages of SBL, which include improved skill acquisition, realistic practice, and flexible learning opportunities; (2) Challenges in Implementation, such as resource constraints, lack of faculty training, and scheduling conflicts; (3) Institutional Factors, including the influence of accreditation and limited policy support; (4) Personal Motivation, highlighting the role of individual faculty initiative; (5) Instructional Approach, focusing on student-centered and structured learning strategies; and (6) Reflective Practice and Continuous Improvement, emphasizing iterative feedback and structured debriefing for enhanced learning outcomes.

**Conclusion:**

The participants valued simulation-based education for its ability to enhance skill acquisition and provide a safe learning environment, but resource constraints, lack of faculty training, and insufficient institutional support hinder its implementation. Addressing these barriers is essential to fully realizing its educational potential.

## INTRODUCTION

Since disease states were taught using clay and stone models hundreds of years ago, simulation has been employed in medical education. Medical schools currently strongly emphasize imparting scientific information, a century after the publication of the initial Flexner report, which has set standards for medical education across the US.^1^ In addition to professional qualities like empathy, compassion, and integrity, there is a need to focus on teaching other crucial abilities, including clinical decision-making, non-technical skills, and clinical reasoning. The best way to get around this is to teach students on actual patients.

The growing concerns regarding patient safety in medical education and training challenge this approach.^2^ In simulation, learners can experience and learn through reliable scenarios to attain the targeted objectives and/or outcomes.^3^ Medical simulation is used worldwide to train and educate students by exposing them to real-world medical scenarios without putting patients at risk.^4^ This offers an alternative to teaching over real patients. Simulation-based medical education has been reported as cost-effective, enhancing performance, and reducing human errors in healthcare delivery.^5^

Another advantageous feature of simulation is the capacity to receive rapid, immediate input. Studies found that using simulators to teach improved student learning outcomes for diagnosis and treatment.^6^ Medical simulation is used in assessment processes more frequently than teaching. Today, medical simulation is underutilized in teaching and training our undergraduates and many specialties. 7 Given the evident benefits of medical education, its application across all healthcare specialties is now more justifiable with proper management.^8^ In the majority of developed countries, healthcare professionals have embraced simulation. While the implications are similar in limited resources settings, countries with limited resources are still having difficulty implementing it widely.^9^

There are many misconceptions about the use of simulation-based education, including its affordability, magnitude, and expenditure, along with doubts about its credibility and integrity.^10^ Still, there are challenges to the use of simulation in medical training. According to a study by Ahmed et al., the high cost of simulators and the shortage of skilled simulation professionals are the main obstacles to simulation use.^11^ Furthermore, it was noted by Al-Ghareeb & Cooper that modern simulators and simulation time limits are obstacles to simulation use.^12^ These obstacles are noted from various research situations and backgrounds, but from the standpoint of the medical faculty, it might be otherwise. A study conducted in Saudi Arabia revealed that faculty members possessed limited knowledge of simulation. They were inexperienced enough to take advantage of the opportunities that simulation offered.^13^ The cost of pricey equipment and staff training was the main barrier to the adoption of simulation.^14^ Regarding simulation deployment in resource-poor contexts, unit cost and operator skills continue to be significant areas of worry.^15^ However, low-fidelity simulators such as Simulated Patients can be quickly implemented, cheap, and widely available.^16^ Medical schools have been pushed to incorporate simulation into their curricula due to the advantages that come with using it in healthcare settings.^4^

There are many studies that explore the attitudes of students undergoing simulation sessions. Still, there are limited studies on the perceptions of benefits and challenges faced by the faculty who are directly involved in operating these sessions and who will incorporate these simulation-based activities into the curriculum. This made the core rationale for this project. Additionally, given the sociocultural dynamics of the region and the constraints of resource-limited settings, there was a gap in the effective understanding of the novel simulation methodologies and their implications. This could revolutionize medical education in regions where clinical exposure is limited and enhance the quality of healthcare delivery.

## METHODOLOGY

This qualitative case study probed the perceptions and experiences of medical faculty about simulation-based education during April - September of 2024. The data were collected from ten medical faculty members of three medical colleges of Khyber Pakhtunkhwa.

### Ethical Approval:

After Advanced Studies and Research Board (AS&RB) approval at Khyber Medical University in June, 2024 (No: DIR/KMU-AS&RB/PO/002809 Dated: September 24, 2024), ethical approval was taken from the KMU-IHPER Ethical Board (Ref No: 1-12/IHPER/MHPE/KMU/24-06 Dated: June 14, 2024).

### Data collection:

A purposive sampling method was used based on their availability and willingness to participate.

### Inclusion & Exclusion criteria:

It included faculty members with at least five years of teaching experience and actively involved in simulation-based teaching to undergraduate students.

Any faculty member who expressed unwillingness to participate in the study was excluded. Semi-structured interviews were employed as the primary data collection tool to gather rich, qualitative insights from faculty members.

The questionnaire used in the interviews was adapted from the Consolidated Framework for Implementation Research (CFIR).^17^ This framework encompasses five key components: intervention characteristics, outer setting, inner setting, characteristics of the individuals involved, and the process of implementation. The initial version of the questionnaire was piloted with four faculty members to assess its clarity, relevance, and cultural appropriateness. Feedback from this pilot testing led to modifications that better suited the sociocultural context of the participants.

Before the in-depth interviews, the refined questionnaire was shared with the faculty members. The interviews were conducted either face-to-face, via phone calls, or via recorded voice SMS, depending on the participants’ preferences, geographical location, and availability. Before starting each interview, participants were required to sign a written informed consent form, which allowed for the tape recording of the sessions. The anonymity and confidentiality were confirmed to the study participants before the interviews. After that, the transcripts were made anonymous before discussing them with co-authors for data analysis. To ensure reflexivity, the research team consisted of two university faculty members, each possessing expertise in simulations and qualitative research methodologies. The remaining researchers were MHPE scholars and had prior experience teaching within the MBBS program. All team members had varying levels of experience with qualitative interviewing techniques to ensure consistency and rigor in data collection. Moreover, the research team practiced bracketing setting aside their preconceptions and biases, focusing on the participants’ perceptions and experiences. To ensure bracketing, enhance rigor, and promote deeper reflection, the interviewers avoided sharing personal information with participants beyond their academic qualifications and the stated purpose of the study. Data triangulation was incorporated to enhance the credibility and reliability of findings through interviews and observations of participants. The process involved member checking of transcripts, ensuring that participants confirmed the accuracy of their responses, and included reviews by supervisors and medical educationists to achieve consensus on themes. Thematic analysis was then used, and all authors agreed on themes and subthemes after a manual thematic analysis was completed. Reflective journals were reviewed during analysis to monitor emerging biases, including researchers’ own experiences with simulation-based education. Instances where assumptions surfaced were discussed in team meetings and documented in the audit trail.

### Data analysis:

The study data transcription was performed manually to minimize the risk of errors and ensure that the nuances of the participants’ responses were preserved. Once transcription was complete, the data was securely stored in Microsoft Excel, providing an organized framework for further analysis. The analysis involved coding and thematic analysis, performed manually to identify key themes. As the analysis progressed, data was further categorized into sub-codes based on the specific responses provided by the study participants. This nuanced coding allowed for a deeper understanding of the data and facilitated the identification of finer distinctions within each main domain. The codes and themes were shared with the study participants to look for their accuracy and resonance with their experiences. The data triangulation was done among study participants and authors to avoid any misinterpretation. The transferability of the findings is supported by the study’s diverse sample.

## RESULTS

This study aimed to explore how faculty members perceive SBE as a tool for enhancing medical students’ clinical skills and competencies and to identify the barriers that affect its implementation and integration within the curriculum. The results include demographics, themes, subthemes, and descriptive quotes from the participants’ interviews.

### Demographics:

Out of 10 participants, six were females (60%) and four were males (40%) as shown in Table-I.

**Table-I:** Demographics of the participants.

Variable	Characteristics	n	%
Gender	Male	4	40
	Female	6	60
Institute of the faculty member	KMC	4	40
	GMC	3	30
	WMC	3	30
Qualifications	MBBS	10	100
	FCPS	8	80
	MHPE	4	40
	PhD	1	10
Designations	In-Charge Skills Lab	2	20
	Professor	1	10
	Assistant Professor	5	50
	Demonstrators	4	40
Departments	Medical Education	3	30
	Surgery	2	20
	Cardiology	1	10
	Medicine	2	20
	Gynecology	2	20
Teaching Experience	>20 years	2	20
	15-20 years	1	10
	10-15 years	2	20
	5-10 years	5	50

### Thematic Analysis:

A total of six themes with seventeen subthemes emerged after the data analysis (Table-II). The study addressed various aspects related to Simulation-Based Learning (SBL).

**Table-II:** Perceptions of Benefits, Challenges, and Utility of Simulation-Based Education among Medical Faculty of Khyber Pakhtunkhwa, Pakistan.

Themes	Subthemes	Participant’s quotations
Advantages of Simulation-Based Learning	Skill Acquisition	- “*Students can learn basic life support skills in remote areas.” **(P#1)***
		*- “Allows unlimited practice on task trainers enabling students to learn from mistakes through repetition without fatigue.” **(P#9)***
		*- “Provides a safe environment where learners can make mistakes without risking patient safety.” **(P#7)***
	Realistic Practice	*- “High-fidelity simulations replicate medical scenarios like cardiac arrest with real-time physiological responses enhancing practical skills under pressure.” **(P#6)***
		*- “High-fidelity simulations using AI for adaptive learning provide realistic scenarios for skill application.” **(P#8)***
	Immediate Feedback	*- “Real-time feedback allows learners to adjust actions and reinforces learning through immediate response.” **(P#5)***
		*- “Real-time feedback helps students adjust their techniques instantly, fostering better retention and skill accuracy.” **(P#10)***
	Flexible and Adaptive Learning	*- “Students can learn anytime according to their own schedule.” **(P#3)***
		*- “Use of various online platforms for adaptive self-directed learning.” **(P#2)***
	Safe Learning Environment	*- “Provides a risk-free environment for learners to experiment and learn from errors without real-world consequences.” **(P#5)***
		*- “Allows students to experiment and learn from mistakes without real-world consequences enhancing experiential learning.” **(P#8)***
Challenges in Implementation	Resource Constraints	*- “The adoption is impeded by financial resource limitations at the administrative level.” **(P#1)***
		*- “Maintaining simulation labs and equipment like VR systems can be expensive limiting accessibility for larger student groups.” **(P#6)***
		*- “High-fidelity simulation models and advanced technology are often expensive, limiting access for students.” **(P#10)***
	Lack of Faculty Training	*- “We do not have skilled motivated teaching staff for mannequin-based training.” **(P#1)***
		*- “Lack of equipment trained and dedicated staff to conduct simulation activities.” **(P#2)***
		*- “Faculty members feel simulation is complex and are less motivated for training and practice.” **(P#4)***
	Institutional Support Gaps	*- “Activities are implemented without set protocols or standards.” **(P#2)***
		*- “Lack of standardized protocols and policy support from institutional leaders limits the effective implementation of simulation-based learning.” **(P#10)***
	Scheduling Conflicts	*- “Limited time for simulation activities due to clinical and lecture schedules.” **(P#2)***
		*- “Faculty engagement is hindered by a lack of motivation and busy clinical schedules limiting participation in simulation training.” **(P#9)***
Institutional Factors	Policy Makers and Accreditation Influence	*- “External requirements from accreditation bodies and safety initiatives influence SBL adoption to improve competencies without patient risk.” **(P#6)***
		*- “Adoption is influenced by accreditation standards external funding and industry benchmarks aligning with broader professional expectations.” **(P#8)***
	Limited Policy Maker Engagement	*- “Policy makers show limited interest with the Dean being the primary motivator for a future skill lab.” **(P#4)***
		*- “Policymakers lack commitment viewing simulation activities as fulfilling regulatory requirements rather than educational enhancement.” **(P#7)***
Personal Motivation	Faculty Initiative and Support	*- “Simulation activities are designed individually by faculty.” **(P#2)***
		*- “Strong personal motivation and positive student response drive the adoption of simulation activities despite minimal institutional support.” **(P#9)***
	Self-Efficacy and Alignment with Organizational Goals	*- “Personal motivation, self-efficacy, and alignment with organizational goals enhance commitment to simulation implementation.” **(P#5)***
		*- “Sense of ownership in institutional goals increases commitment to implementing simulations effectively and aligning with educational objectives.” **(P#8)***
Instructional Approach	Student-Centered and Adaptive	*- “Instructional strategies are modified based on undergraduate student feedback.” **(P#2)***
		*- “Instructional methods are adapted based on feedback from colleagues and students emphasizing flexibility and student-centered learning.” **(P#9)***

	Structured Scenario Design	*- “Each simulation phase from design to self-reflection is structured to reinforce learning and facilitate continuous improvement.” **(P#8)***
		*- “Systematic stages from preparation to evaluation support simulation effectiveness ensuring realistic scenarios and immediate feedback.” **(P#5)***
Reflective Practice and Continuous Improvement	Iterative Feedback Mechanisms	*- “Feedback from simulation activities is used to refine tools and approaches enhancing the effectiveness of each session.” **(P#9)***
		*- “Faculty and students collaboratively refine simulation models and procedures based on ongoing feedback enhancing overall learning outcomes.” **(P#10)***
	Reflective Debriefing	*- “Structured debriefing sessions allow learners to critically reflect on their performance.” **(P#6)***
		*- “Dr. XYZ facilitates structured debriefing sessions to allow students to reflect on their actions, discuss errors, and solidify their learning experience.” **(P#10)***

- ***Theme-1:*** The advantages of SBL indicated that participants appreciated the secure environment it offers, facilitating repeated practice without jeopardizing patient safety, while high-fidelity simulations permitted complex scenario training supplemented by quick feedback for real-time improvements.
- ***Theme-2:*** Challenges in Implementation highlighted obstacles like higher equipment costs, the necessity for specialized faculty training, and time limitations arising from competing clinical responsibilities.
- ***Theme-3:*** Institutional Impact indicated that certification standards, policy formulation, and quality assurance mechanisms are pivotal in influencing adoption and sustainability of simulation.
- ***Theme-4:*** Personal Factors emphasized the significance of faculty initiative, dedication, and continuous professional growth in fostering successful initiatives.
- ***Theme-5:*** The instructional approach emphasized organized preparation, experiential learning via practical engagement, and performance-focused evaluation as essential pedagogical components.
- ***Theme-6:*** Reflective Practice revealed that iterative feedback, reflective debriefing, and candid discussions augment session efficacy, foster critical self-reflection, and reinforce learning results.

## DISCUSSION

Our findings reveal key benefits, challenges, and essential factors shaping simulation-based learning (SBL) in medical education. Faculty members identified several advantages, such as enhanced skill acquisition through repeated, risk-free practice and realistic simulations that prepare students for complex scenarios like trauma and cardiac arrest. SBL’s flexibility and accessibility, even in low-resource settings, along with immediate feedback during simulations, were highly valued as they strengthened learning outcomes. However, barriers like financial constraints, insufficient faculty training, and limited institutional policy support hinder broader adoption.

Additionally, faculty-driven motivation, where faculty members were committed to continue their simulation teaching without any reward or praise but their internal will and self-race for excellence, and structured reflective practices were found to be crucial for fostering an effective, student-centered, and continuously improving learning environment. Our participants noted that simulation-based learning (SBL) enhances skill acquisition by allowing practice in a safe environment without risking patient harm. This aligns with Ajmi et al., who found that SBL revealed critical skill gaps in procedures like donning and doffing, which wouldn’t have been easily identified through traditional assessments.^18^ Elshami et al. also found that SBL allowed students to confidently apply learned skills in clinical practice, with 69% of students reporting successful implementation in real-world scenarios.^19^

Implementing SBL often encounters barriers such as resource limitations, lack of faculty training, and insufficient institutional support. Vera et al. reported that financial constraints hindered the scalability of SBL in critical care, particularly in under-resourced settings.^20^ Additionally, Kuchyn et al. observed that although simulation improved students’ clinical skills, the lack of standardized protocols and trained faculty prevented comprehensive implementation.^21^ Park et al emphasized the need for developing real scenarios simulating clinical environments and highlighted the challenges of costs, resource limitations, and technical barriers.^22^ These findings resonate with our participants’ concerns about the high costs of maintaining simulation labs and the need for dedicated faculty training to support SBL initiatives.

The effectiveness and sustainability of SBL are often influenced by institutional policies and external accreditation requirements. Owen et al. demonstrated that simulation-based Advance Care Planning (ACP) training boosted healthcare professionals’ confidence, a result driven by accreditation demands rather than intrinsic educational goals.^23^ Our findings align with this perspective, as participants noted that policymakers often view simulation as a regulatory formality rather than a learning enhancement. Mileder et al. observed similar challenges, indicating that policy engagement beyond compliance is necessary for SBL sustainability.^24^ Faculty members’ motivation is crucial for driving and sustaining SBL initiatives, particularly when institutional backing is limited. Our findings showed that individual faculty initiatives often keep SBL alive within institutions. This is consistent with Katoue et al., who found that educators’ commitment played a central role in implementing simulation despite limited institutional support.^25^

Our study highlighted the importance of adaptive, student-centered instructional strategies in SBL. Participants noted that instructional methods are frequently modified to meet student needs, emphasizing flexibility and scenario-based learning. Meerdink et al. supported this approach, finding that healthcare professionals benefitted more from simulations using actors (simulated patients) over manikins due to the higher realism and adaptability to learner feedback.^26^ Goolsarran et al. also showed that structured instructional methods improved learning outcomes and teamwork in simulation-based patient safety training.^27^ Reflective practice and continuous feedback emerged as key components for effective SBL. Our participants emphasized the importance of structured debriefing sessions for learners to analyze and improve their performance. This mirrors findings from Hencklein et al. who noted that clinical simulations with built-in reflective practices greatly enhanced students’ self-confidence and knowledge.^28^ A systematic method must be used when adopting simulation as a teaching strategy in healthcare programs for students of all ages. The development of skillful staff is the starting point. The next stage is to include simulation into an educational course’s standard coursework. This can be accomplished by creating novel programs independently or by incorporating this form of instruction into the current curriculum and to incorporate technological advancements of simulation-based learning in teaching strategies to improve students’ academic and clinical training quality.^29^-^32^

### Limitations:

Accessing faculty directly involved in simulation-based teaching proved challenging, despite the presence of skills labs and active sessions. Support staff, primarily responsible for operational facilitation, were unable to refer us to appropriate participants and declined involvement themselves. Several faculty members declined interviews, often citing time constraints or feeling unqualified due to limited theoretical knowledge of simulation. Notably, none of the participants had received formal training in simulation-based education, relying instead on experiential practice.

## CONCLUSION

The perceived benefits and uses of simulation-based education (SBE) among medical faculty include enhanced skill acquisition, realistic practice through high-fidelity simulations, and the provision of immediate feedback that improves learning outcomes. Faculty members emphasized that SBE creates a safe learning environment, allowing students to practice clinical skills without risking patient safety, which is particularly beneficial in resource-limited settings.

Significant challenges encountered by medical faculty in the use and implementation of SBE include resource constraints, insufficient faculty training, scheduling conflicts, and a lack of institutional support, which hinder effective integration into the curriculum. Participants noted that while SBE offers substantial educational advantages, the barriers to its implementation must be addressed to maximize its potential benefits for medical education.

There is a need for enhanced institutional policies and support systems that facilitate the effective adoption of simulation-based education, ultimately improving the quality of training for future healthcare professionals.

### Future Recommendations:

Future studies should explore a broader range of participants, including different demographics, professions, or regions, to enhance the generalizability of the findings. Conducting longitudinal research can provide deeper insights into how the identified themes evolve over time and influence outcomes in the long run. Collaboration with multiple disciplines can enrich understanding by integrating perspectives from dentistry, allied health sciences and other relevant fields. Exploring the role of emerging technologies in relation to the themes discussed could offer innovative solutions and practical applications. Translating research findings into actionable policies or interventions will ensure that the study contributes to real-world improvements. Comparing findings across different cultural or organizational contexts can uncover unique insights and further validate the study’s conclusions.
